# Behavioral characteristics of autism spectrum disorder in very preterm birth children

**DOI:** 10.1186/s13229-019-0282-4

**Published:** 2019-07-22

**Authors:** Li-Wen Chen, Shan-Tair Wang, Lan-Wan Wang, Yu-Chia Kao, Ching-Lin Chu, Chin-Chin Wu, Yi-Ting Hsieh, Chung-Hsin Chiang, Chao-Ching Huang

**Affiliations:** 1 Department of Pediatrics, National Cheng Kung University Hospital, College of Medicine, National Cheng Kung University, 138 Sheng-Li Road, Tainan, 70403 Taiwan; 2 Graduate Institute of Clinical Medicine, College of Medicine, National Cheng Kung University, Tainan, Taiwan; 3 Graduate Institute of Gerontology, College of Medicine, National Cheng Kung University, Tainan, Taiwan; 4 Department of Pediatrics, Chi-Mei Medical Center, Tainan, Taiwan; 5 Department of Pediatrics, College of Medicine, Taipei Medical University, Taipei, Taiwan; 6 Department of Biotechnology, Southern Taiwan University of Science and Technology, Tainan, Taiwan; 7 Department of Pediatrics, E-Da Hospital, Kaohsiung, Taiwan; 8 Department of Educational Psychology and Counseling, National Pingtung University, Pingtung, Taiwan; 9 Department of Psychology, Kaohsiung Medical University, Kaohsiung, Taiwan; 10 Department of Psychiatry, National Cheng Kung University Hospital, College of Medicine, National Cheng Kung University, Tainan, Taiwan; 11 Department of Psychology, National Chengchi University, Taipei, Taiwan; 12 Research Center for Mind, Brain and Learning, National Chengchi University, Taipei, Taiwan

**Keywords:** Autism spectrum disorder, Preterm, Very low birth weight, Autism Diagnostic Observation Schedule, Autism Diagnostic Interview-Revised

## Abstract

**Background:**

Lower gestational age may increase autism spectrum disorder (ASD) vulnerability; however, the incidence of ASD diagnosis through a direct assessment on every very preterm birth child on the population base remains unclear. Moreover, the behavioral characteristics of preterm birth ASD are unknown.

**Methods:**

Every very preterm birth child (gestational age < 32 weeks; birth weight < 1500 g) who was discharged from neonatal intensive care units in Southern Taiwan and prospectively followed to 5 years of age was evaluated using the Autism Diagnostic Observation Schedule (ADOS) and the Autism Diagnostic Interview-Revised (ADI-R). The term birth (gestational age > 37 weeks) ASD children characterized by ADOS and ADI-R were group matched to the preterm birth ASD by age at examination for comparison. ADOS severity scores were calculated by the Mann–Whitney *U* test and ADI-R by multivariate analysis of variance and canonical discriminant analysis.

**Results:**

Two hundred forty-six (87%) of the 283 very preterm survivors were followed prospectively to 5 years of age. Nineteen (7.7%) of the 246 children fulfilled the diagnostic criteria of ASD. After excluding 1 patient with cerebral palsy and profound mental disability, 18 preterm ASD children were compared with 44 term birth ASD children. The two ASD groups were comparable for age at examination, gender, and intelligence quotient. The two groups showed comparable ADOS severity scores in social affect deficits, restricted repetitive behaviors, and total score, but had differences in qualitative abnormalities in reciprocal social interaction (Wilks lambda *F* value = 6.2, *P* < 0.001) of ADI-R. Compared to term birth ASD children, preterm birth ASD children exhibited worse nonverbal behaviors that regulate social interaction (OR 2.59, 95% CI 1.41–4.73, *P* = 0.002) but more favorable peer relationships (OR 0.58, 95% CI 0.38–0.90, *P* = 0.01) and socioemotional reciprocity (OR 0.55, 95% CI 0.33–0.92, *P* = 0.02). In contrast to the heterogeneous severity of social reciprocity in the term ASD group, the behavioral characteristics of the preterm ASD group showed a homogeneous reciprocal social interaction pattern.

**Conclusions:**

The 5-year incidence rate of ASD was high in very preterm birth children. Preterm birth ASD exhibited a specific behavioral phenotype of reciprocal social interaction.

**Electronic supplementary material:**

The online version of this article (10.1186/s13229-019-0282-4) contains supplementary material, which is available to authorized users.

## Background

Autism spectrum disorder (ASD) is a neurodevelopmental disorder characterized by social communication difficulties and a restricted repetitive behavioral pattern presenting during young childhood [1]. The etiology of ASD is complex. Genetic factors could explain some disorders; however, nongenetic components, for example, pregnancy complications, drug exposure during pregnancy, environmental factors, and preterm birth, may also play important roles [2–5]. Large-scale ASD studies have mainly focused on children in the general population, who are mostly born at term, while relatively few studies have focused on the low gestational age preterm infants.

Advances in neonatal intensive care have greatly improved the survival rate of very preterm infants; however, the incidence of neurodevelopmental impairments in these infants is a major concern [6]. Severe neurodevelopmental disabilities, such as cerebral palsy and sensory impairments, show a declining trend. Additionally, socioemotional and behavioral problems in the surviving preterm birth children have received more attention due to impacts on school achievement and mental health [6–8]. Recent studies have demonstrated that preterm infants have a higher prevalence of ASD, as a shorter gestational duration is associated with greater vulnerability [9–12]. Because preterm infants have a 3–4 times higher risk of ASD than term infants in the general population, ASD screening has been emphasized during neurodevelopmental follow-up programs for children born preterm [9, 11]. There is a discrepancy between screening and diagnosis of ASD in the preterm population due to confounding factors from brain injury and neurological sequela frequently associated with preterm birth [13]. In addition, limited studies have performed direct ASD assessments prospectively on every child in the population-based very preterm birth cohort.

Distinct from most infants born at term, preterm infants experience more severe complications during their perinatal and neonatal periods. Several factors, including prematurity per se, may increase the risk of ASD in the preterm population [9, 11, 14, 15]. Despite the known prevalence and risk factors, symptom severity and phenotypic characteristics of preterm birth ASD children, relative to term birth ASD children, are still unclear. A previous study found that the preterm birth males with ASD tended to have more comorbidities such as seizure disorders, sleep apnea, and attention-deficit/hyperactivity disorder than did term birth males with ASD [16]. However, whether or not there are differences in ASD symptoms, such as social communication difficulties and restricted repetitive behaviors, remains unknown.

Characterization of the ASD phenotype could be important for clinical services and research purposes. For measuring core ASD features, Autism Diagnostic Observation Schedule (ADOS) via behavioral observation and Autism Diagnostic Interview-Revised (ADI-R) via caregiver interview are the most extensively studied among all diagnostic tools [17]. Utilizing direct and universal examination by ADOS and ADI-R on every very preterm child at age 5 years, this prospective follow-up population study in Southern Taiwan investigated the following: (1) the incidence rate of ASD in very preterm population and (2) the behavioral characteristics and autism symptom severity of the preterm birth ASD children in comparison to the term birth ASD children.

## Methods

### Preterm population

In total, 283 very preterm infants (birth weight < 1500 g; gestational age < 32 weeks) who survived to discharge from the 4 neonatal intensive care units of Tainan City, a southern city in Taiwan with a population of 1,880,000, from 2008 to 2013 were enrolled. Two hundred forty-six of the 283 infants (a follow-up rate of 87%) were followed prospectively at a special clinic in the university hospital up to 5 years of age. This clinic provides neurodevelopmental and autism assessments for every very preterm birth child at 5 years of age [18, 19]. The mean gestational age of this 5-year-old preterm cohort was 28 ± 2 weeks, the mean birth weight 1066 ± 248 g, and the gender ratio (male vs. female) of 1.3.

### ASD patient groups

The preterm birth children who were diagnosed with ASD (preterm ASD group) in the cohort were matched by age at examination with term birth (gestational age > 37 weeks) children with ASD (term ASD group). The term birth ASD children were recruited from the ASD special clinics at the university hospital and from the university ASD study center from August 2015 to June 2018. This study was approved by the university hospital’s institutional review board.

### Neurodevelopmental and ASD assessments

The sites for ASD evaluation were supervised by Professor CH Chiang, an ASD specialist who received research training and research certification in the USA. He trained with Dr. Catherine Lord’s team in Michigan and Dr. Catherine Rice at the Centers for Disease Control and Prevention [20, 21]. Regarding Autism Diagnostic Interview-Revised (ADI-R), Dr. CH Chiang got the researcher certificate for reliability in December 2003 from Dr. Christopher Smith in Mount Sinai School of Medicine, New York. In terms of Autism Diagnostic Observation Schedule (ADOS), Dr. Chiang had received clinician and research trainings with Dr. C. Lord’s group (Drs. Christina Corsello and Amy Esler) in June 2004. He was then approved by Dr. C. Rice for ADOS research certificate for reliability when he collaborated with Drs. C. Rice and Li-Ching Lee to host an ADOS workshop for clinicians and researchers at Pingtung, Taiwan, in February 2008.

Child psychologists evaluated the cognitive function of the two ASD groups by using the Wechsler Preschool and Primary Scale of Intelligence, Fourth Edition (WPPSI-IV), or the Wechsler Intelligence Scale for Children, Fourth Edition (WISC-IV), and Mullen Scales of Early Learning [22]. Intellectual disability was defined as a full-scale intelligence quotient or a developmental quotient less than 70. Cerebral palsy was diagnosed on the basis of abnormal muscle tonicity and limitations in gross motor function with a score greater than 2.

A team that included pediatric neurologists, child psychiatrists, and child psychologists established the ASD diagnosis through comprehensive diagnostic assessments on the two groups of children. This diagnosis was made on the basis of Diagnostic and Statistical Manual of Mental Disorders, 5th edition, ADI-R, and ADOS, First Edition [23, 24]. The ADOS is a semi-structured assessment of behavioral observation, in which developmentally appropriate social and play-based interactions in a 40- to 60-min session are designed to elicit autism symptoms. The ADI-R is a semi-structured standardized parent interview developed to assess the presence and severity of autism symptoms throughout childhood. The combination of ADOS and ADI-R is considered the gold standard for ASD diagnosis in research [17]. In this study, the rater was not blind to the perinatal history including the gestational age of the child at examination. There was initial diagnostic uncertainty in 2 preterm birth children, and a team meeting was held to discuss these cases to make the final diagnoses (Additional file 1 Supplementary methods).

Different modules of ADOS were chosen according to the children’s developmental status, ranging from those with no expressive language to fluent verbal expression [25]. To standardize ASD severity across the different modules of ADOS, an algorithm was applied to the raw data of two domains (social affect deficits and restricted repetitive behaviors) and a total score composed of social affect deficits and restricted repetitive behaviors domains was calculated [26, 27]. The ASD symptom profile was represented by the variables of ADI-R subscores [28, 29]. The general severity of ASD symptoms was analyzed by comparing ADOS severity scores, and the ASD symptom profiles were generated by applying discriminant analysis on ADI-R.

### Statistical analysis

Differences in the demographics of the preterm ASD and term ASD groups were compared using Fisher’s exact test for categorical variables, and for numerical variables, Mann–Whitney *U* test was performed due to non-normal distribution in most of the data. Standardized ADOS severity scores were compared using the Mann–Whitney *U* test. Scores of each ADI-R domain were evaluated using multivariate analysis of variance (MANOVA). Canonical discriminant analysis was applied to identify a linear combination of ADI-R variables with the most discriminatory power between the preterm ASD and term ASD groups. Multiple variable selection procedures for binary logistic regression, including stepwise, best subset, and all possible subset selection methods using information criteria, were performed. The resulting optimal subset for prediction was identified and analyzed, and Firth approximation was conducted for small-sample adjustment [30]. Bootstrap sampling was conducted to evaluate the reliability of the selected subset of variables. Subsequently, the classification accuracy of the combination of ADI-R variables was challenged via leave-one-out cross-validation. The significance level was set at *P* < 0.05 for 2-sided hypothesis tests. Bonferroni’s correction and false discovery rate (FDR) were applied to adjust for multiple comparisons [31]. Post hoc power analysis on sample size determination for two-group linear discriminant analysis was also performed [32]. The post hoc power analysis suggested that the minimum group size for minimum overlap should be larger than 14 for various prevalence rates. Statistical analysis was performed using SAS software and GraphPad Prism 5.

## Results

Among the 246 very preterm birth children who completed the autism assessments at 5 years of age, 19 subjects met the ASD diagnostic criteria, yielding a 5-year ASD incidence of 7.7% in this very preterm population. The ADI-R and ADOS severity scores of the 227 preterm-birth children who did not have ASD were presented in Additional file 1: Table S1. One preterm child, who fulfilled the ASD diagnostic criteria, was excluded from the analysis due to concomitant multiple handicaps including severe cerebral palsy and profound intellectual disability. Forty-four term birth children with ASD were matched by age at examination with the 18 very preterm birth children with ASD. In the preterm ASD group, the mean age was 62 months (SD [standard deviation] 4 months) at examination, and the mean intelligence quotient was 83 (SD 16; range 62–108). Four children (22%) in the preterm ASD group displayed intellectual disability (Table 1). The mean age of the term ASD group was 64 months (SD 16 months) at examination, with a mean intelligence/developmental quotient of 79 (SD 23; range 34–113). Fifteen children (34%) in the term ASD group had intellectual disability. The preterm ASD group had significantly lower mean gestational age and mean birth weight than did the term ASD group (Table 1). One patient (6%) in the preterm birth group had epilepsy, and two patients (5%) in the term birth group had epilepsy. There was no observed congenital abnormality in either group. Both groups were comparable in gender, age at examination, intelligence/developmental quotient, and the percentage of intellectual disability.

**Table 1:** Demographic differences between the preterm birth and term birth children with autism spectrum disorder (ASD)

Variable	Preterm ASD *n* = 18	Term ASD *n* = 44	*P* value
Gestational age, mean (SD), weeks	28 (2)	39 (1)	< 0.001
Birth body weight, mean (SD), grams	1024 (267)	3196 (391)	< 0.001
Age of examination, mean (SD), months	62 (4)	64 (16)	0.25
Male, *n* (%)	12 (67%)	38 (86%)	0.09
Intellectual disability, *n* (%)	4 (22%)	15 (34%)	0.54
Intelligence quotient, mean (SD); range	83 (16); 62–108	79 (23); 34–113	0.66

The phenotypic characteristics were compared across various behavioral aspects in the ADI-R between the two ASD groups. Symptoms defined by the ADI-R involve qualitative abnormalities in reciprocal social interaction (domain A) and communication (domain B); restricted, repetitive, and stereotyped behavioral patterns (domain C); and abnormality in development evident at or before the age of 36 months (domain D). Each of the domains A, B, and C was composed of 4 subscores, correlating to specific ASD symptoms. According to the results of the MANOVA, qualitative abnormalities in reciprocal social interaction (domain A) were significantly different between the preterm ASD and term ASD groups (Wilks lambda *F* value = 6.2, *P* < 0.001) (Table 2). By contrast, no differences were observed between the two groups in qualitative abnormalities in communication (domain B; Wilks lambda *F* value = 1.5, *P* = 0.20) and in restricted, repetitive, and stereotyped behavioral patterns (domain C; Wilks lambda *F* value = 1.3, *P* = 0.28). After applying Bonferroni’s correction and FDR to adjust for multiple comparisons, A2, which represents failure to develop peer relationships, was the most significant discriminatory variable.

**Table 2:** Autism Diagnostic Interview-Revised (ADI-R) score differences between the preterm and term ASD groups

ADI-R item	ADI-R score^a^		MANOVA			Bonferroni’s correction	FDR
	Preterm *n* = 18	Term *n* = 44	*F* value	*P* value	Wilks lambda	*P* value	*P* value
Domain A: Qualitative abnormalities in reciprocal social interaction					*F* value = 6.2 *P* < 0.001		
A1: Failure to use nonverbal behaviors to regulate social interaction	3.8 (1.9)	3.2 (1.8)	1.7	0.20		1	0.34
A2: Failure to develop peer relationships	3.9 (2.0)	5.6 (1.8)	10.2	0.002		0.024	0.024
A3: Lack of shared enjoyment	3.2 (1.8)	3.7 (2.0)	0.9	0.35		1	0.47
A4: Lack of socioemotional reciprocity	4.1 (2.0)	5.2 (2.3)	3.2	0.08		0.96	0.19
Total A: A1 + A2 + A3 + A4	14.9 (6.5)	17.6 (6.1)	2.3	0.13			
Domain B: Qualitative abnormalities in communication					*F* value = 1.5 *P* = 0.20		
B1: Lack of or delay in spoken language and failure to compensate through gesture	2.9 (2.4)	3.9 (2.4)	1.4	0.24		1	0.36
B4: Lack of varied spontaneous make-believe or social imitative play	3.6 (1.7)	4.0 (1.5)	0.2	0.65		1	0.71
B2(V)^b^: Relative failure to initiate or sustain conversational interchange	2.8 (1.2)	3.4 (1.1)	3.5	0.07		0.84	0.19
B3(V)^b^: Stereotyped, repetitive, or idiosyncratic speech	2.4 (2.0)	3.4 (1.9)	3.1	0.08		0.96	0.19
Total B(V)^b^: B1 + B4 + B2(V) + B3(V)	11.8 (5.4)	14.4 (4.3)	3.8	0.06			
Domain C: Restricted, repetitive, and stereotyped behavioral patterns					*F* value = 1.3 *P* = 0.28		
C1: Encompassing preoccupation or circumscribed pattern of interest	1.2 (1.4)	1.8 (1.2)	2.9	0.10		1	0.2
C2: Apparently compulsive adherence to nonfunctional routines or rituals	1.2 (1.5)	1.4 (1.5)	0.1	0.73		1	0.73
C3: Stereotyped and repetitive motor mannerisms	0.8 (1.1)	1.0 (1.0)	0.5	0.49		1	0.59
C4: Preoccupation with parts of objects or nonfunctional elements of material	1.0 (0.8)	1.5 (0.8)	4.3	0.04		0.48	0.19
Total C: C1 + C2 + C3 + C4	4.2 (3.1)	5.6 (3.0)	2.8	0.10			

*MANOVA* multivariate analysis of variance, FDR false discovery rate

^a^Data are expressed as mean (SD)

^b^Scoring only in children with verbal ability

Canonical discriminant analysis and binary logistic regression were applied to identify significant subscores within domain A and to calculate the performance index for discrimination. All possible subset selection methods using Akaike information criteria and Bayesian information criteria suggested enrollment of A1, A2, and A4. The results of stepwise selection and resulting binary logistic regression using Firth approximation were presented in Table 3. Higher scores in the ADI-R represent more severe symptoms; thus, the preterm ASD group had worse nonverbal behaviors to regulate social interaction (A1, odds ratio 2.59, 95% CI [confidence interval] 1.41–4.73, *P* = 0.002) but showed more favorable development of peer relationships (A2, odds ratio 0.58, 95% CI 0.38–0.90, *P* = 0.01) and superior socioemotional reciprocity (A4, odds ratio 0.55, 95% CI 0.33–0.92, *P* = 0.02) than did the term ASD group. The multivariate regression model composed of A1, A2, and A4 could discriminate the preterm ASD group from the term ASD group, with an area under the receiver operating characteristic curve of 0.86 (95% CI 0.77–0.95) (Additional file 1: Figure S1).

**Table 3:** Multiple logistic regression analysis of qualitative abnormalities in reciprocal social interaction (domain A in ADI-R) in the preterm and term ASD groups

ADI-R item	Estimate	SE	OR	95% CI	*P* value
Failure to use nonverbal behaviors to regulate social interaction (A1)	0.95	0.31	2.59	1.41–4.73	0.002
Failure to develop peer relationships (A2)	− 0.55	0.22	0.58	0.38–0.90	0.01
Lack of socioemotional reciprocity (A4)	− 0.59	0.26	0.55	0.33–0.92	0.02

*ADI-R* Autism Diagnostic Interview-Revised, *CI* confidence interval, *OR* odds ratio, *SE* standard error

**Fig. 1:**
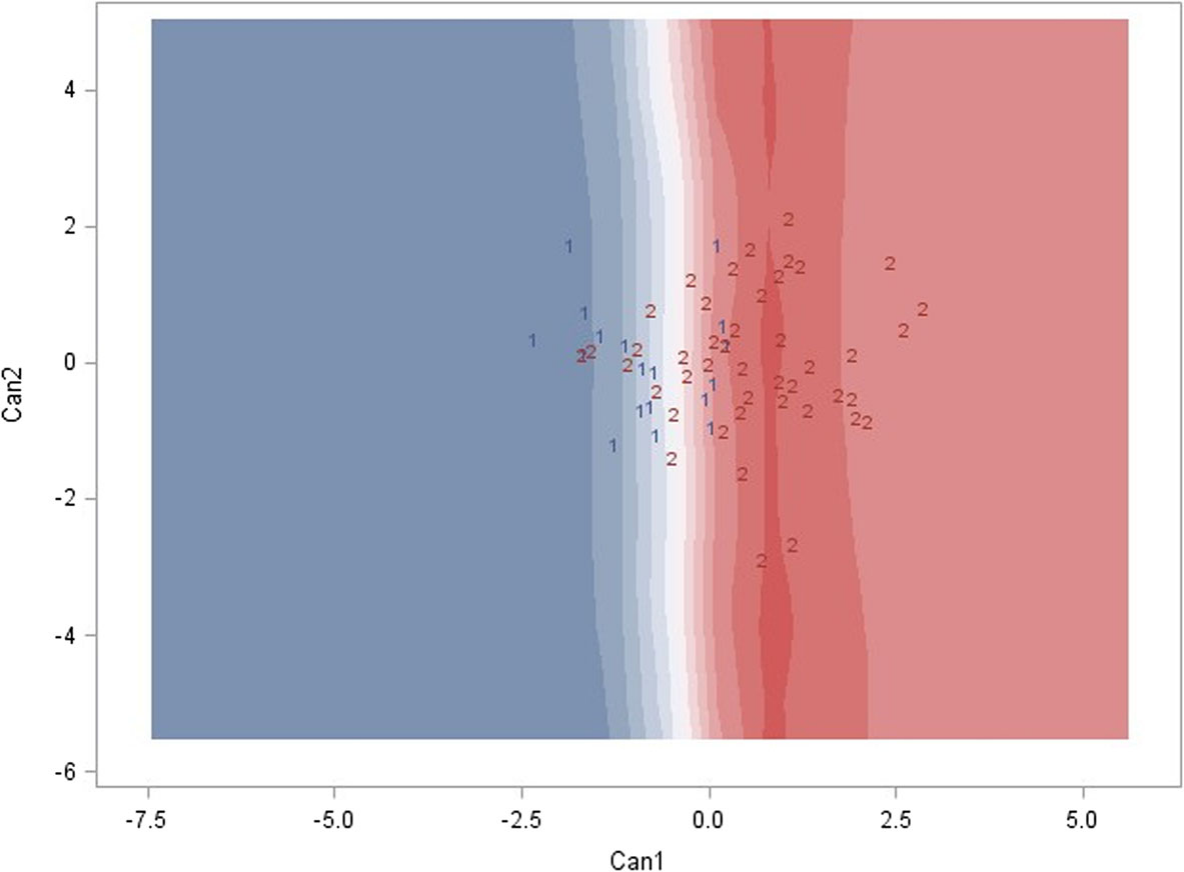
Canonical discriminant analysis for preterm birth and term birth ASD by ADI-R. According to A1 (failure to use nonverbal behaviors to regulate social interaction), A2 (failure to develop of peer relationships), and A4 (lack of socioemotional reciprocity) in ADI-R, canonical discriminant analysis revealed an aggregated distribution of preterm birth ASD (labeled as 1 in blue) and a broad distribution of term birth ASD (labeled as 2 in red)

According to canonical discriminant analysis, the behavioral characteristics of the term ASD group showed a broad spectrum across the dimension composed of A1, A2, and A4, representing the heterogeneous severity of reciprocal social interaction (Fig. 1). By contrast, the behavioral characteristics of the preterm ASD group had a clustered distribution, representing the homogeneous reciprocal social interaction pattern.

The severity of ASD symptoms was compared between the two ASD groups after transforming ADOS raw data into severity scores in social affect deficits and restricted repetitive behaviors domains and the total score. One patient in the term ASD group was excluded because only the overall ADOS ratings were available and severity scores could not be calculated. Comparison of ADOS severity scores showed no significant differences in social affect deficits, restricted repetitive behaviors, or the total score between the two ASD groups (Additional file 1: Figure S2). Therefore, we concluded the overall ASD severity was comparable between these two groups according to ADOS severity scores.

## Discussion

The risk of ASD may be increased with lower gestational age [9, 14]; however, few studies have performed direct and universal diagnostic examinations without prior screening procedures in every very preterm birth child. In our prospective population-based study in Southern Taiwan, diagnostic assessments with ADOS and ADI-R revealed an overall 5-year ASD incidence rate of 7.7%. The preterm and term birth ASD children showed no differences in gender ratio, age at examination, or the percentage of intellectual disability. The two ASD groups were comparable in the ASD symptom severity by ADOS severity scores but demonstrated differences in the quality of social reciprocity according to ADI-R. The preterm ASD children showed worse nonverbal behaviors to regulate social communication but exhibited more favorable peer-relationships development and socioemotional reciprocity than the term ASD children. The preterm ASD children could be discriminated from the term ASD children by their behavioral characteristics. In contrast to the heterogeneous severity of reciprocal social interaction in the term ASD group, the behavioral characteristics of the preterm ASD group had a clustered distribution, representing the homogeneous reciprocal social interaction pattern. These findings of specific behavioral pattern may suggest a unique difference in the brain networking for the social interaction phenotype between the preterm and term birth children with ASD.

Previous studies in preterm infants showed that the positive rates of ASD by screening tests are extraordinarily high, ranging from 21 to 26% [33, 34]. However, false positivity resulting from concomitant neurodevelopmental deficits indicates the need for precise differential diagnosis in the high-risk preterm population [13]. Considering the limitations of ASD screening tests in preterm population, we conducted both ADOS and ADI-R examinations on every very preterm child in the population cohort in Southern Taiwan and found a 5-year ASD incidence rate of 7.7%. Even after excluding the preterm child with major neurological disabilities, the ASD incidence rate was still high at 7.3%. The ASD incidence rate found in this very preterm population study correlated to the 7% prevalence rate of ASD in a meta-analysis study of children born preterm [10]. The high incidence of ASD in preterm population might be caused by preterm birth per se and/or by alteration of immature brain growth during the development from smooth cortex to the gyrated cortex during extra-uterine life [35–37]. In addition, as genetic factors may play a role in both preterm birth and ASD, common candidate genes involving the two aspects could also contribute to the final consequence of preterm birth ASD [38]. More investigations are required to test these possible hypotheses, such as the effect of shared genetic pathway of preterm birth and ASD on the disturbed maturation process of immature brains.

Measuring ASD severity across various behavioral dimensions enables comparison of clinical assessments [26]. In the present study, the ADOS severity scores of social affect deficits, restricted repetitive behaviors, and the total score were comparable between the preterm ASD and term ASD groups. Therefore, our results demonstrate similar severity of behavioral symptoms between the preterm and term birth ASD children according to the clinical observation of ADOS.

Based on the caregiver’s observation in ADI-R, compared to term ASD group, the preterm birth children with ASD had a particular pattern of reciprocal social interaction (domain A in the ADI-R). They showed both favorable and worse outcomes in distinct aspects of social reciprocity. The preterm birth children with ASD exhibited worse nonverbal behaviors in social interaction (A1), such as direct gaze, social smiling, and communicative facial expressions, but showed more favorable peer relationships (A2), including imaginative play, interest in children, response to other children’s approach, and group play with peers. The preterm ASD group also showed superior socioemotional reciprocity (A4), for example, less atypical behavior of using another’s body to communicate, better presentation of offering comfort, and preferable quality and appropriateness of social responses compared to the term ASD group. Although strength was observed in the preterm ASD children, studies reveal that adults with very preterm birth tend to have global withdrawn personality [39]. How the social interaction deficits impact long-term personality, especially when they have better social motivations than term birth ASD at childhood, should be a concern for the possible developmental mechanism and for the targeted interventions for preterm population.

The phenotypic differences observed in ADI-R but not in ADOS may reflect the value and limitation of each tool. Both ADOS and ADI-R measure the core ASD symptoms; ADOS shows a high diagnostic accuracy during a short period of clinical observation, and ADI-R complements the diagnosis by obtaining a semi-structured interview of caregiver history [40]. ADI-R has an advantage of being more comprehensive with behavioral patterns provided by the caregiver, which may not be measured in the time-limited observation of ADOS. Therefore, some items of reciprocal social interaction, such as peer relationships, may be better demonstrated in ADI-R. Although ADI-R has been used for phenotype subgrouping and symptom profiling in ASD research, the discrepancy between ADI-R and ADOS is also present in previous studies [28, 29]. According to studies on the agreement of ADI-R and ADOS algorithms, ADI-R and ADOS were shown to have independent and additive contributions to ASD diagnosis, due to low correlations between the two diagnostic tools [41]. As a result, the phenotypic differences of reciprocal social interaction according to ADI-R, but not from ADOS severity scores, may be attributed to the measurement issues of the two diagnostic tools.

According to the characteristics in “failure to use nonverbal behaviors to regulate social interaction (A1)”, “failure to develop peer relationships (A2)”, and “lack of socioemotional reciprocity (A4)” in the ADI-R, we found that the term ASD group showed heterogeneous behavioral symptoms across the dimension of reciprocal social interaction, whereas the preterm ASD group showed rather clustered distribution in this dimension. These findings reflected the nature of heterogeneous profiles in the term birth ASD children, but a more homogeneous phenotype in the preterm birth ASD children that might be categorized as a subgroup within the broad ASD spectrum.

Several neurobiological mechanisms may underlie the characteristic behavioral symptoms of ASD. For example, gaze fixation during facial discrimination task may be related to fusiform gyrus-amygdala connections, and social reward behaviors could be correlated to frontal-striatal circuits [42–44]. Our findings of worse nonverbal behaviors including direct gaze and facial expression, and more favorable peer relationships and socioemotional reciprocity in preterm ASD group might suggest the different neuro-circuits involvements between the term birth and preterm birth children of ASD. Additional functional magnetic resonance neuroimaging studies comparing the preterm birth and term birth children with ASD could help to identify the unique differences in neurobiological substrates between the two ASD groups.

Our study has several limitations. The social reciprocity phenotypes in children at 5 years of age were analyzed in our study because the behavioral pattern may be more stable, and the differential diagnosis of various developmental problems could be easier than in young toddlers [45]. However, as the intervention window closely relates to the early identification of ASD, conducting ASD diagnosis at a younger age should be considered in the very preterm cohort. There were several factors confounding the collection and interpretation of ADI-R and ADOS. Because ADI-R is performed through diagnostic interviews with the main caregiver, recall bias may exist. It was possible that the parents of the preterm birth ASD children might interpret and report their children’s symptoms differently from that of the term birth ASD group, leading to reporting bias [46, 47]. On the other hand, as the term birth ASD children visited the clinics due to parents’ concerns, the parents may have more definite observations of behaviors. As a result, the difference in the recruitment of the two ASD groups could have biased the results. Therefore, the consistency of behavioral presentations should be further evaluated in different situations, such as interviewing teachers at school. Although there was no significant difference in gender between the two groups, the percentage of female was slightly higher in the preterm group. Since gender might affect the perception of behaviors, we cannot exclude the possibility of gender influence on the phenotype analysis at long-term follow up [16, 48–50]. The study is limited for the small sample size of ASD in children who are very preterm birth, even though we did a population-based ASD assessment on most very preterm child in southern Taiwan. Although the data was corrected for multiple comparisons, the interpretation of the phenotypes should be cautious. As a result, multi-center studies with a larger sample size and a priori power analysis are required to verify the behavioral phenotypic characteristics of very preterm birth children with ASD, as shown in this study. Despite the limitations, our findings suggest that preterm ASD may be considered as a subgroup of specific social reciprocity phenotype, which can provide a window for delineating the unique neurobiological basis and for implementing a suitable intervention considering both the favorable and worse outcomes of preterm-birth children with ASD.

## Conclusions

Diagnostic evaluations revealed a high incidence rate of ASD in the very preterm population. Compared with the term ASD children, the preterm ASD children had more favorable peer relationships and socioemotional reciprocity but showed worse nonverbal behaviors that regulate social communication. These findings suggest ASD children with preterm birth may be a unique subgroup of the ASD and the neuro-circuitry involved in the social interaction may underlie the difference in the neurobiological substrate between the two groups of ASD children.

## Additional file

Additional file 1: Supplementary methods. **Table S1.** Evaluations of the preterm birth children without autism spectrum disorder (ASD). **Figure S1.** Discrimination between the preterm and term ASD groups by ADI-R. **Figure S2.** ADOS severity scores of preterm birth and term birth ASD. (DOCX 7516 kb)

## Data Availability

The dataset used and analyzed during the current study are available from the corresponding author on reasonable request.

## Abbreviations

ADI-R: Autism Diagnostic Interview-Revised
ADOS: Autism Diagnostic Observation Schedule
ASD: Autism spectrum disorder
CI: Confidence interval
FDR: False discovery rate
MANOVA: Multivariate analysis of variance
SD: Standard deviation
WISC-IV: Wechsler Intelligence Scale for Children, Fourth Edition
WPPSI-IV: Wechsler Preschool and Primary Scale of Intelligence, Fourth Edition
