# Supplementary material for: Behavioral characteristics of autism spectrum disorder in very preterm birth children
Source: Mol Autism. 2019 Jul 22;10:32. doi: 10.1186/s13229-019-0282-4 (PMC6647137; doi:10.1186/s13229-019-0282-4)

**Supplementary Methods.**

Two preterm birth children who had diagnostic uncertainty were discussed in the joint team meeting. One was classified as ASD, but was excluded from phenotype analysis due to severe multiple handicaps, including cerebral palsy and profound intellectual disability. Another one was found to have neurofibromatosis type 1 and was classified as non-ASD with cognitive developmental delay.

**Table S1.** Evaluations of the preterm birth children without autism spectrum disorder (ASD).

| **Variable** | **Preterm non-ASD (n = 227)** |
| --- | --- |
| Age of examination, mean (SD), months | 61 (2) |
| Intellectual disability, n (%) | 17 (7%) |
| Intelligence quotient, mean (SD); range | 89 (13); 43–121 |
| **ADI-R** |  |
| Total A: Qualitative abnormalities in reciprocal social interaction, mean (SD); range | 1.6 (2.4); 0-18 |
| Total B (verbal): Qualitative abnormalities in communication, mean (SD); range | 0.6 (1.7); 0-14 |
| Total C: Restricted, repetitive, and stereotyped behavioral patterns, mean (SD); range | 0.1 (0.7); 0-10 |
| **ADOS severity score** |  |
| Social affect deficits, mean (SD); range | 1.7 (1.3); 1-9 |
| Restricted repetitive behaviors, mean (SD); range | 2.0 (2.0); 1-9 |
| Total score, mean (SD); range | 1.5 (1.1); 1-8 |

**Figure S1. Discrimination between the preterm and term ASD groups by ADI-R.** According to A1 (failure to use nonverbal behaviors to regulate social interaction), A2 (failure to develop of peer relationships), and A4 (lack of socioemotional reciprocity) in ADI-R, the preterm birth ASD could be discriminated from the term birth ASD with area under the receiver operating characteristic curve 0.86 (95% CI: 0.77-0.95).


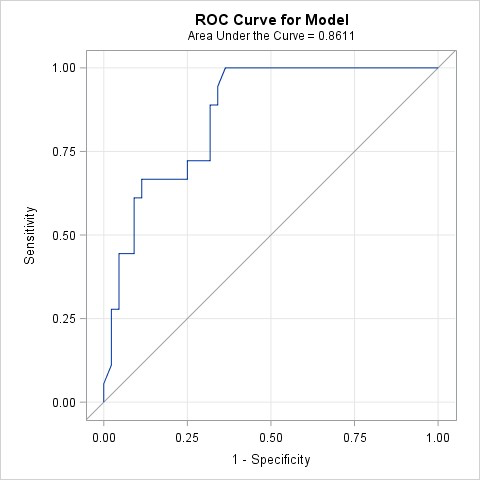


**Figure S2. ADOS severity scores of preterm birth and term birth ASD.** No differences were observed in the social affect deficits (SA, *P* = 0.84), restricted repetitive behaviors (RRB, *P* = 0.62), and the total score (*P* = 0.80).


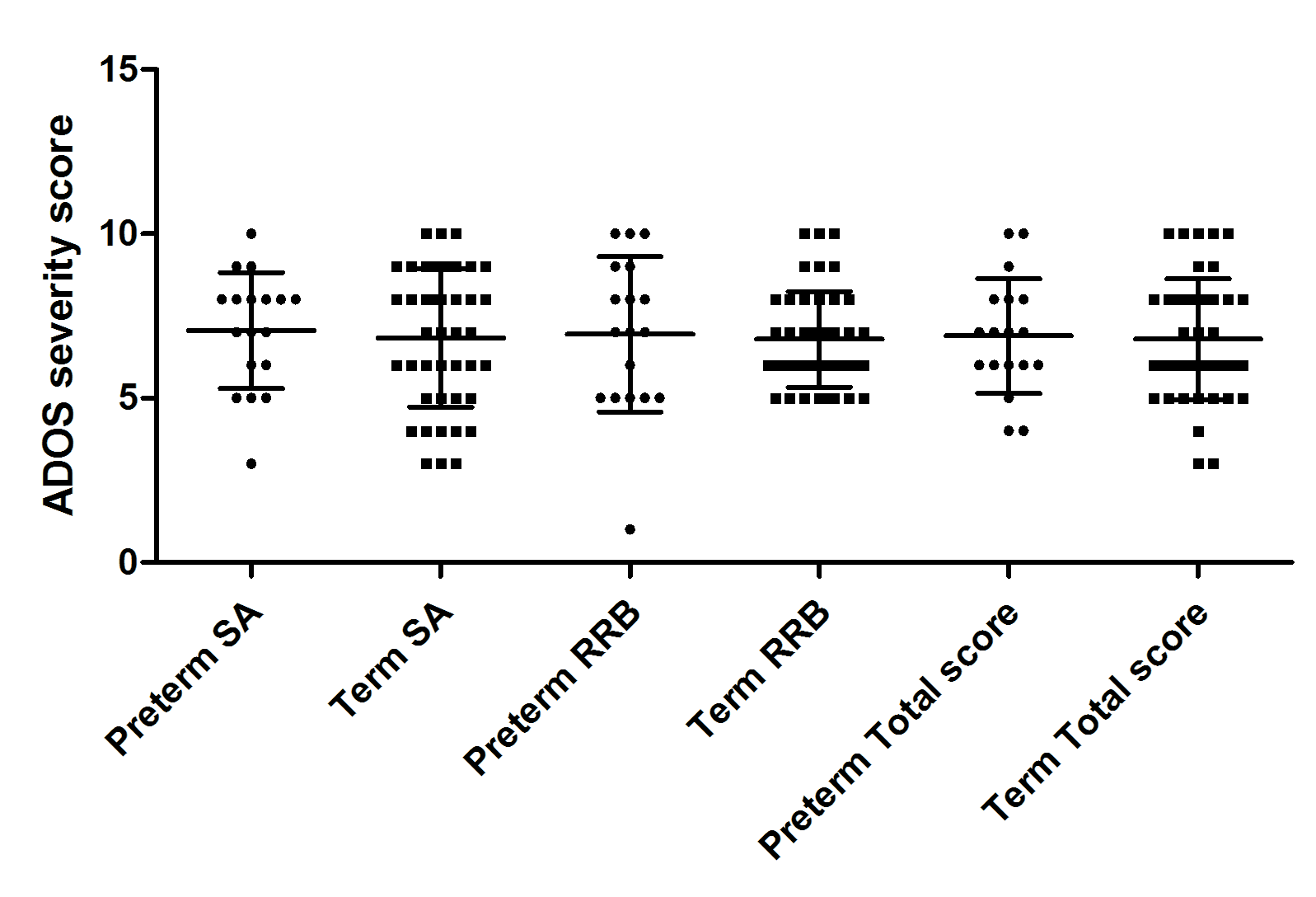

Supplement: Supplementary file 1 — Supplementary methods. Table S1. Evaluations of the preterm birth children without autism spectrum disorder (ASD). Figure S1. Discrimination between the preterm and term ASD groups by ADI-R. Figure S2. ADOS severity scores of preterm birth and term birth ASD. (DOCX 7516 kb) [file 13229_2019_282_MOESM1_ESM.docx]
